# Postoperative Pain Control with the Fentanyl Patch and Continuous Paravertebral Anesthetic Infusion after Posterior Occipitocervical Junction Surgery

**DOI:** 10.7759/cureus.645

**Published:** 2016-06-17

**Authors:** Walavan Sivakumar, Michael Karsy, Andrea Brock, Richard H Schmidt

**Affiliations:** 1 Department of Neurosurgery, Clinical Neurosciences Center, University of Utah

**Keywords:** posterior fossa surgery, occipitocervical, chiari decompression, pain control, fentanyl

## Abstract

Postoperative pain is a significant concern for patients who undergo surgery via a midline posterior approach to the occipitocervical junction and spinal axis. The development of the disposable, ambulatory pain pump presents a novel alternative for treatment of postoperative pain. The authors describe a multimodal treatment algorithm for postoperative pain after posterior occipitocervical junction surgery that uses the On-Q pain catheter system (I-Flow Corp., Lake Forest, CA) and a fentanyl patch.

The On-Q PainBuster catheter system is a disposable, ambulatory device that allows for continuous anesthetic delivery directly into or adjacent to the wound. On-Q catheters are placed in the nuchal musculature for continuous infusion of 0.5% bupivacaine. The On-Q catheter infusion is continued for three days, and the catheters are then withdrawn. Patients are also provided with a transdermal fentanyl patch at the start of surgery.

In regards to complications at our facility, there have been no cases of respiratory depression or arrest postoperatively and no wound infections, but one case of inadvertent subdural placement.

The technique described for the use of the fentanyl patch and a continuous anesthetic delivery device in surgery of the occipitocervical junction presents a novel alternative to the current standard of care in pain control after suboccipital decompression.

## Introduction

Postoperative pain is a significant concern for patients who undergo surgery via a midline posterior approach to the occipitocervical junction and spinal axis. Most of the pain is due to extensive soft-tissue and muscle dissection, along with intraoperative manipulation [1]. Debilitating pain after surgery can result in increased opiate requirements, delayed ambulation, prolonged hospital length of stay, and significant postoperative morbidity [2]. The current standard of care requires a multimodal treatment approach involving opioid narcotics, nonsteroidal anti-inflammatory drugs, benzodiazepines, and patient-controlled anesthesia devices [1]. The advent of the disposable, ambulatory pain pump presents a novel alternative for treatment of postoperative pain [3]. Continuous anesthetic delivery devices have been in use at our institution in general surgery, vascular surgery, orthopedic spine surgery, and occipitocervical junction and spine surgery since 2009. The efficacy of continuous postoperative local anesthetic in decompression surgery for the occipitocervical junction has not been evaluated previously. Here, we describe our multimodal treatment algorithm for postoperative pain after posterior occipitocervical junction surgery using the On-Q pain catheter system and a fentanyl patch.

## Technical report

### On-Q pain catheter system

**Figure 1:**
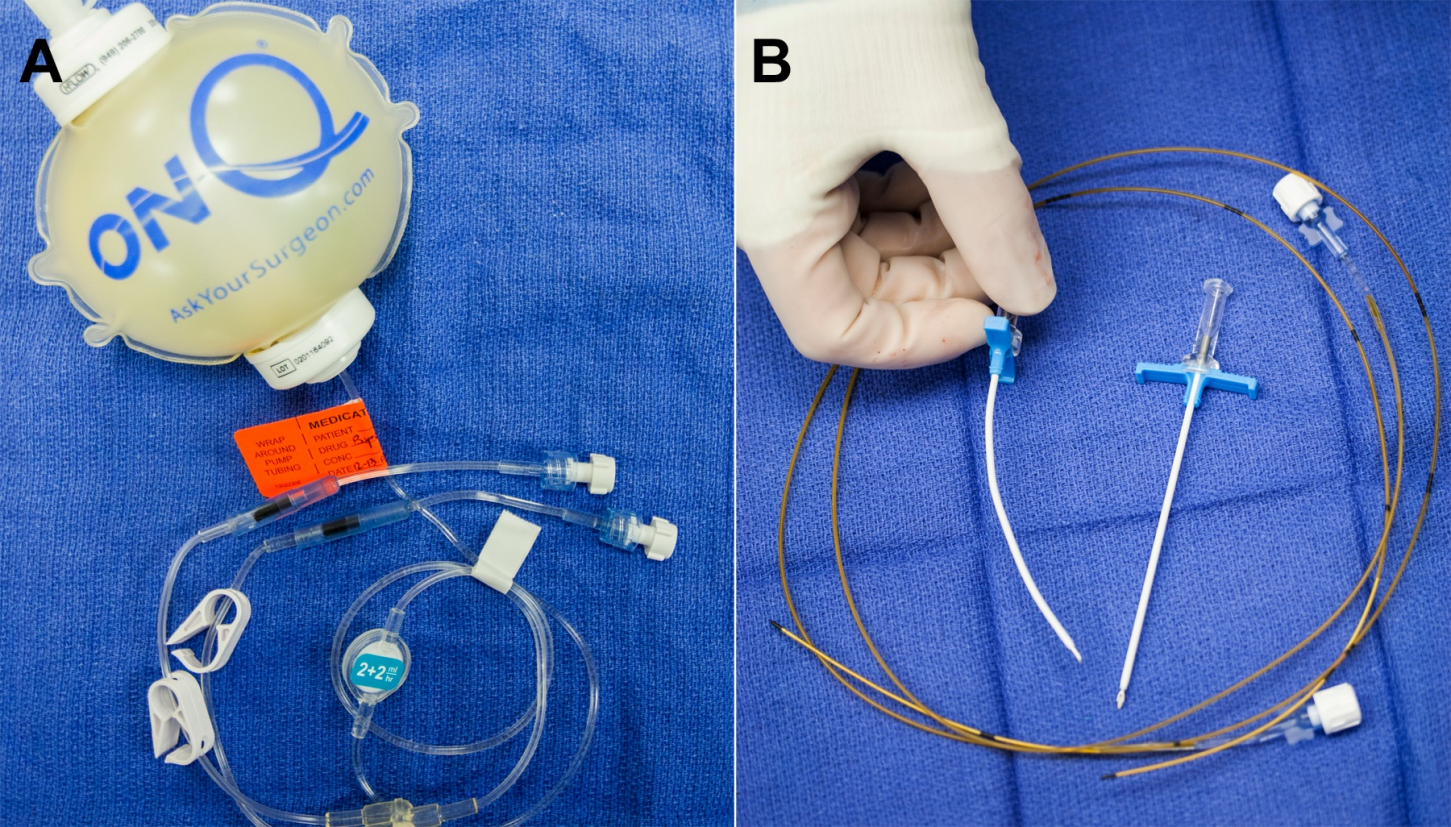
Photograph showing the On-Q PainBuster system. The On-Q PainBuster system consists of (A) an elastomeric pump containing 0.5% bupivacaine with tubing and flow-restrictors and (B) two 20-gauge infusion catheters with trocars and T-handle introducer sheaths.

The On-Q PainBuster (I-Flow Corp., Lake Forest, CA) catheter system is a disposable, ambulatory device that allows for continuous anesthetic delivery directly into or adjacent to the wound. This device (Figure 1) utilizes an elastomeric pump and a flow restrictor to maintain a continuous and consistent drug elution at a pressure of 10 lb/in^2^. The pumps exist in various volumes and flow rates. One or two catheters of different lengths may be attached to the pump [4]. At our institution, 0.5% bupivacaine is the anesthetic of choice.

### Intraoperative management

All patients undergoing posterior fossa cranial surgeries are given dexamethasone during anesthesia induction (4–8 mg), and steroids are continued postoperatively on a tapering schedule to mitigate meningeal headache. Patients are also provided with a transdermal fentanyl patch at the start of surgery, and this is continued perioperatively until the patch is exhausted. Adult opiate-naïve patients are given a 25-μg/h transdermal patch, whereas those using narcotics preoperatively are given enough additional fentanyl to cover this baseline requirement, up to a 100-μg/h transdermal patch.

**Figure 2:**
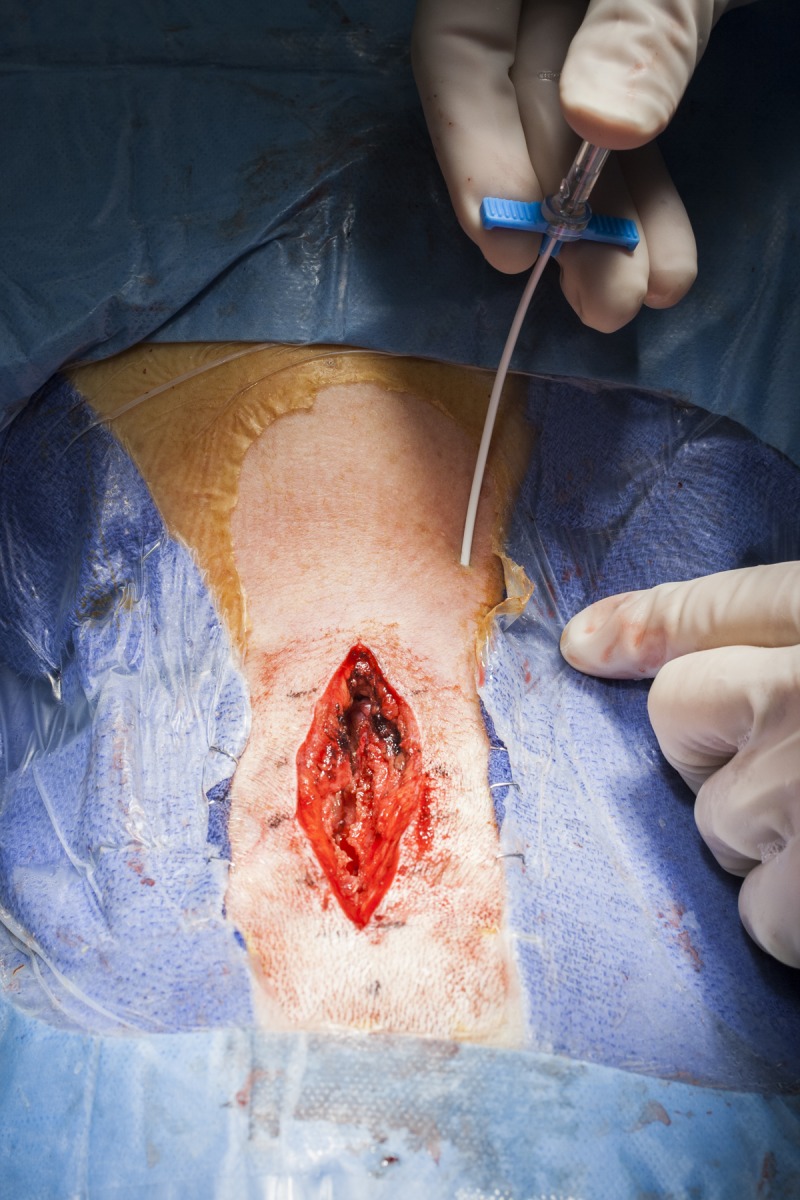
Intraoperative photograph showing the insertion of the introducer sheath and trocar, which have been curved to follow the curve of the neck. The sheath is inserted approximately 1.5 – 2 cm lateral to the incision at a depth of 2 cm. Concurrent neck and finger palpation is used to guide the catheter.

**Figure 3:**
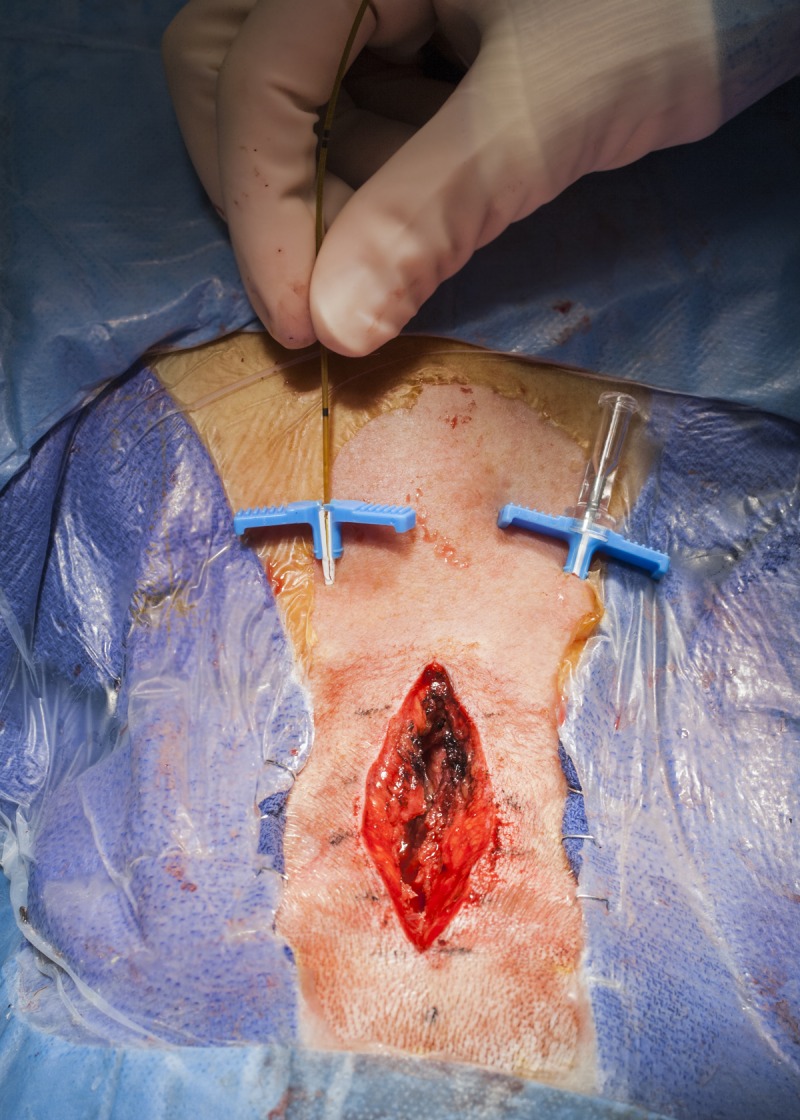
Intraoperative photograph showing the insertion of the 5-cm infusion catheters, which are passed through the introducer sheath after the trocar is removed.

Once the main part of the operation is concluded and the dura is closed, On-Q catheters are placed in the nuchal musculature for continuous infusion of 0.5% bupivacaine. For patients with a midline occipitocervical wound similar to that used for Chiari decompression, 5-cm infusion catheters are placed at the start of wound closure via percutaneous insertion parallel to and approximately 1.5–2 cm lateral to the incision and coursing within the nuchal musculature at a depth of approximately 2 cm. The introducer sheaths can be curved to follow the curve of the neck, and finger palpation within the wound can be used to guide the introducer sheath within the course of the musculature (Figure 2). Care is taken to avoid catheter entry into the surgical incision, and extreme care must be taken to avoid dural penetration with the introducer sheaths or catheters. The catheters are then passed through the introducer sheaths in the standard fashion (Figure 3). Once in place, the catheters are secured with stay sutures at the entry site. Once the catheters are placed, 5 ml of 0.25% bupivacaine is infused into each catheter. Also at this time, patients are given 2.5 mg of intravenous (IV) diazepam to mitigate postoperative muscle spasm. At the conclusion of surgery, the catheters are connected to the elastomeric pump and infusion is started. All catheters are taped securely with translucent adhesive dressings to prevent pull out and allow inspection of the insertion site.

### Postoperative management

Patients who have undergone a posterior fossa craniotomy via a midline approach are managed initially in the neurocritical care unit for 24 hours to allow monitoring for any sign of respiratory depression or impairment of consciousness. Breakthrough surgical pain is managed initially with IV fentanyl and IV acetaminophen, and patients are transitioned to oral narcotics as tolerated (hydrocodone or oxycodone), usually within 12–24 hours. The fentanyl patch remains in place during this time, and cervical muscle spasm is managed with IV or oral diazepam and topical heating pads or ice. Steroids are tapered over a four to seven day period as tolerated. Patients are typically mobilized on postoperative day 1 and transferred to the regular nursing floor. The On-Q catheter infusion is continued for three days at a continuous and consistent drug elution, and the catheters are then withdrawn. Should a dressing change be required, it is performed in a sterile fashion.

### Results

To date, this protocol has been implemented in 60 patients undergoing suboccipital craniotomies with or without C1 laminectomies for Chiari decompressions over a period of 34 months. No patients required postoperative orthotic support. We are now in the process of a complete retrospective collection and analysis of this data, assessing the degree of pain relief and satisfaction in the On-Q catheter fentanyl patch protocol group compared with those treated prior to the On-Q catheter fentanyl patch protocol. There have been no cases of respiratory depression or arrest postoperatively and no wound infections. Early in our experience, there was one case of inadvertent catheter insertion subdurally in which a loading dose (5 ml) of 0.25% bupivacaine was administered. The On-Q catheter was placed in a caudal to rostral direction. This patient remained deeply anesthetized for one hour postoperatively requiring continued ventilatory support, and there was a transient loss of all brainstem reflexes, including nonreactive dilated pupils, but no hemodynamic instability. After one hour, the patient awakened and recovered fully with no residual impairment. Postoperatively, the wound was monitored for a CSF leak, and there was no evidence of a pseudomeningocele or clinical evidence of intracranial hypotension. The risk of this complication can be avoided if the depth and trajectory of catheter insertion are carefully controlled and also if a rostral-caudal insertion path is used. The most common problem with the On-Q system in this application has been premature pull-out of one or more catheters or significant percutaneous drug leakage at the insertion site, requiring earlier than intended catheter removal.

The University of Utah Institutional Review Board issued approval IRB_00051346 for the retrospective chart review, which is exempt from further IRB review under Exemption Category 7.

## Discussion

Postoperative pain is a common and significant concern for patients undergoing posterior midline occipitocervical surgery. Debilitating pain after surgery can result in an excessive opiate requirement and its subsequent sequelae—nausea, vomiting, urinary retention, constipation, delayed mobilization, decreased oral intake, and delayed discharge [2]. The technique described above presents a novel alternative to the current standard of care in pain control after suboccipital decompression. Our preliminary experience for postoperative pain control in posterior fossa surgery shows the On-Q infusion device is safe for use with appropriate precautions. A controlled prospective trial will be necessary to confirm its superior efficacy compared with conventional perioperative management, in terms of pain control, earlier mobilization, shorter hospital length of stay, and fewer narcotic-related complications.

Our experience also yielded several important observations. First, improved catheter design would be helpful for this application, specifically to incorporate a better means to secure the device to the scalp above the neck to prevent pull-out and back leak from the insertion site. With conventional taping and use of stay stitches, catheter pull-out was the most frequent limitation in effective utilization. Secondly, special care must be taken in this region to avoid inadvertent catheter insertion into the intradural space. We recommend always inserting from a rostral to caudal direction, away from the posterior fossa, and using visual and manual control within the wound to assure insertion into the center of the paravertebral musculature, above and lateral to the thecal sac. In one early case where inadvertent intradural passage with a catheter placed in a caudal to rostral direction and bupivacaine infusion occurred, no permanent injury resulted, although there was a prolonged emergence from anesthesia. Intraoperative fluoroscopy or X-ray confirmation of catheter placement may be utilized in the event that there is confusion in regards to catheter placement.

Continuous paravertebral anesthetic infusion of bupivacaine has already been shown to decrease postoperative pain and rescue medication usage in lumbar spinal fusion surgery [4-5]. Studies of posterior cervical and lumbar fusion surgeries have shown that pain scores and narcotic pain medication usage decrease without an increase in complications in the first five postoperative days when continuous anesthetic infusion applied directly to the affected paraspinal musculature via an elastomeric pump and flow restrictor is allowed [4-5]. Here, we present the first description of the use of the fentanyl patch and continuous anesthetic delivery device in surgery of the occipitocervical junction and unique considerations that should be taken into account in this region.

## Conclusions

The technique described for the use of the fentanyl patch and a continuous anesthetic delivery device in surgery of the occipitocervical junction presents a novel alternative to the current standard of care in pain control after suboccipital decompression. Its effectiveness in regards to previous pain control regimens will be assessed in a prospective trial.
